# Deep Learning Enhanced Volumetric Photoacoustic Imaging of Vasculature in Human

**DOI:** 10.1002/advs.202301277

**Published:** 2023-08-02

**Authors:** Wenhan Zheng, Huijuan Zhang, Chuqin Huang, Varun Shijo, Chenhan Xu, Wenyao Xu, Jun Xia

**Affiliations:** ^1^ Department of Biomedical Engineering University at Buffalo The State University of New York Buffalo New York NY 14260 USA; ^2^ Department of Computer Science and Engineering University at Buffalo The State University of New York Buffalo New York NY 14260 USA

**Keywords:** 3D vascular Imaging, deep learning, linear transducer arrays, photoacoustic tomography

## Abstract

The development of high‐performance imaging processing algorithms is a core area of photoacoustic tomography. While various deep learning based image processing techniques have been developed in the area, their applications in 3D imaging are still limited due to challenges in computational cost and memory allocation. To address those limitations, this work implements a 3D fully‐dense (3DFD) U‐net to linear array based photoacoustic tomography and utilizes volumetric simulation and mixed precision training to increase efficiency and training size. Through numerical simulation, phantom imaging, and in vivo experiments, this work demonstrates that the trained network restores the true object size, reduces the noise level and artifacts, improves the contrast at deep regions, and reveals vessels subject to limited view distortion. With these enhancements, 3DFD U‐net successfully produces clear 3D vascular images of the palm, arms, breasts, and feet of human subjects. These enhanced vascular images offer improved capabilities for biometric identification, foot ulcer evaluation, and breast cancer imaging. These results indicate that the new algorithm will have a significant impact on preclinical and clinical photoacoustic tomography.

## Graphical abstract

A 3D fully‐dense U‐net is implemented in linear array based photoacoustic tomography. The trained network restores the true object size, reduces the noise level, improves the contrast at deep regions, and addresses the limited view problem. The performance is further validated in various human imaging applications, such as biometric identification, foot ulcer evaluation, and breast cancer imaging.

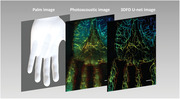

## 1 Introduction

Photoacoustic (PA) tomography (PAT), a hybrid technique combining high optical contrast and deep acoustic penetration, has emerged as a promising medical imaging modality. In PAT, a diffused laser pulse illuminates the region of interest to excite PA waves, which are received by the transducers placed at different locations.^[^ ^1^, ^2^, ^3^ ^]^ To reconstruct an image of the optical deposition, researchers have developed the delay and sum (DAS) algorithm, which back projects raw‐channel data into the imaging domain.^[^ ^4^ ^]^ Among various transducer arrays applied in the PAT, linear transducer arrays are one of the most commonly used due to their low costs and wide clinical adoption.^[^ ^5^, ^6^ ^]^ However, the linear array was designed for two‐dimensional (2D) imaging and its three‐dimensional (3D) imaging performance is poor. For example, the elevation resolution of a linear array is typically a few times worse than its axial and lateral counterparts.^[^ ^7^, ^8^ ^]^ The linear array is also subject to the limited‐view problem, causing certain features to be invisible in the reconstructed image.^[^ ^9^ ^]^ Moreover, electromagnetic interference (EMI) noises are commonly seen in PAT images due to the weak signal and strong laser interferences, which further degrades the linear array PAT performance.^[^ ^10^ ^]^ Addressing these problems would enable wider adoption of linear array based 3D PAT and facilitate its clinical translation.^[^ ^11^, ^12^, ^13^, ^14^ ^]^

Deep learning assisted medical imaging enhancement has seen tremendous progress in the last decade, due to increases in computing power and open‐source networks.^[^ ^10^, ^15^, ^16^, ^17^ ^]^ Several deep learning methods have been proposed in PAT and they have demonstrated promising enhancements.^[^ ^17^, ^18^, ^19^, ^20^, ^21^ ^]^ Notably, a few groups have implemented deep learning in 3D PAT. For instance, Hauptmann et al. utilized 3D images from computerized tomography (CT) scans to train the neural network.^[^ ^22^ ^]^ The algorithm could recover detailed vasculature from under‐sampled data obtained by an optical ultrasound sensor‐based PA system. Guan et al. developed a Dense Dilated UNet for 3D photoacoustic imaging reconstruction in a cylindrical geometry with sparse data sampling.^[^ ^23^ ^]^ Recently, Choi et al. implemented a 3D deep learning approach for a hemispherical transducer array to achieve high frame rate functional 3D PAT with under‐sampled data.^[^ ^18^ ^]^ It can be seen that most of these studies focused on addressing the sparse sampling problem and therefore require a much smaller dataset for training (less than 1500 volumetric images). In addition, the specialized transducer arrays employed in these studies may not directly translate to clinical applications, which typically utilize linear or curve‐linear transducer arrays. As a result, although these studies have shown enhanced image quality, there are limited demonstrations of using the neural network in practical and clinical human applications.

In terms of linear array based deep learning research, Liu. et al. recently proposed a sparse‐sampling reconstruction method to recover the volumetric image acquired from a linear array transducer.^[^ ^24^ ^]^ However, the algorithm focused on sparse sampling instead of resolution improvement and the number of transducer elements used in their study is fewer than most linear arrays. Our group also proposed an algorithm named Deep‐E, which converted the 3D problem into 2D by simulating and training data in the axial‐elevation plane.^[^ ^25^ ^]^ While promising results were demonstrated in human data, the algorithm also has a few limitations. First, the algorithm processes data only in the 2D space without exploring the connection among frames. Therefore, slight misalignments were observed in Deep‐E processed images, making certain vessels look discontinuous. Second, as the 2D space contains fewer features than 3D, underfitting is more likely to happen.^[^ ^26^ ^]^ Finally, the training only considers thermal noise, while the actual system also has EMI noise. In a follow‐up study, we combined Deep‐E with 3D reconstructed data and added experimental noise to the training.^[^ ^27^ ^]^ However, since the training is still performed in 2D, the misalignment between frames still exists.

To overcome these issues, we introduce 3D fully‐dense U‐net (3DFD U‐net) to improve the 3D imaging performance of linear array based PAT. To ensure an accurate simulation of experimental conditions, we implemented several improvements in the study. First, the transducer parameters were precisely defined in 3D to mimic the actual transducer used in the experiment. Second, the simulation volume was defined based on the transducer's receiving aperture to save computing time and memory. Third, to provide effective EMI noise removal, experimental noise was added to simulated data. Finally, 3DFD U‐net was implemented in our deep learning model to produce precise predictions in 3D space. Compared to 3D U‐net,^[^ ^28^ ^]^ 3DFD U‐net can effectively capture more vascular features from 3D PA images because of the extra dense connection provided in the network. Our results demonstrated that our learned model could precisely recover the sizes of objects and improve the continuity of the vascular structure. Meanwhile, the overall image quality was improved as EMI noises and artifacts were greatly removed. Moreover, with reduced noises, vessels from deep regions could be revealed, which is essential for deep‐tissue PAT. In addition, features distorted by the limited‐view problem were also partially recovered in both simulated and in vivo validation. These improvements allow us to bring the algorithm to various human clinical imaging applications.

## 2 Experimental Section

### 2.1 Photoacoustic Imaging System

Details of the imaging system have been described previously in.^[^ ^8^ ^]^ Briefly speaking, we used a compact PAT system where the light illumination and the acoustic detection are coplanar with each other. PA signals were generated by a 10 Hz Nd: YAG Laser (Continuum Inc.) firing 1064 nm output with ≈700 mJ pulse energy and <10 ns pulse width. The output from the laser was coupled to a bifurcated fiber bundle with a 1 cm diameter input and 9 cm line‐shape output. PA signals were detected by a customized 2.25 MHz linear array transducer (IMASONIC SAS) with 128 elements. The transducer was designed to be waterproof with a cubic shape for easy mounting and scanning in water. To improve the detection efficiency, we used two dichroic mirrors to combine acoustic and optic paths into the same plane.^[^ ^5^, ^8^ ^]^ As the line‐shape fiber bundle output and transducer are mounted parallel to each other, this design also improves the system's compactness. The scanning head was mounted on a translational stage (McMaster‐Carr) integrated with a stepper motor for 3D scanning. During scanning, objects were pressed against an imaging window located above the scanning head. Signals from the transducer were digitized by a 14‐bit 256‐channel Data acquisition (DAQ) unit (Verasonic Inc.) and transferred to the host computer for processing. Q‐switch signal output from the laser synchronizes the motor, DAQ, and light delivery.

For human imaging experiments, all studies were approved by the Institutional Review Board of the University at Buffalo, under different study protocols for hand (STUDY00000171), foot (STUDY00001165), and breast imaging (STUDY00000371). Data presented in this study were selected from existing pool of datasets. All human subjects provided informed consent after fully understanding the implications of their participation. Subjects were recruited within the university or by clinical collaborators in the participating clinics. The recruited patients have either confirmed chronic foot ulcer or breast cancer, as indicated by their clinical reports. As the studies were not clinical trials, they were not registered.

### 2.2 Artificial Neural Network

**Figure 1:**
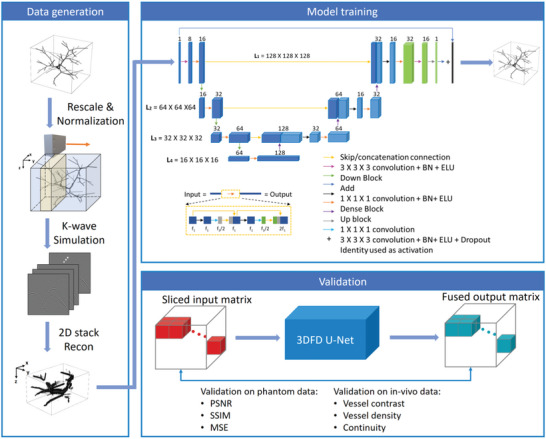
Workflow of the network training procedure. *X*, *Y*, and *Z* in the figure represent lateral, elevation, and axial directions of the transducer, respectively.

As shown in the modal training block of **Figure** **1**, the proposed 3DFD U‐net follows the conventional architecture of the fully dense U‐net,^[^ ^29^, ^30^ ^]^ except that it has 3D kernels. It maintains the encoder and decoder structures to extract the most important features without destroying the shapes of the images and makes up for the losses produced in the encoder path.^[^ ^31^ ^]^ Unlike the 3D U‐net, 3DFD U‐net leverages the benefit of dense connectivity. At the forward propagation steps, the dense block can provide a more diverse set of features with high efficiency. In the dense block, it creates additional feature maps that are generated based on the original input and previously learned features. Moreover, it can avoid the vanishing gradient problem and improve the model performance during the backward propagation steps. During training, the use of concatenation between each convolutional layer enables gradient information to backpropagate to the earlier layers directly. We also exchanged the max pooling layers in the network. They were replaced by 2 × 2 × 2 convolutional layers with a stride of two. It allows the model to learn flexible spatial transformation in the encoder rather than the rigid max pooling procedure.^[^ ^32^ ^]^ RELU activation is exchanged with ELU activation, as it has been shown to improve learning speed in deeper residual networks.^[^ ^33^ ^]^ An additional 3 × 3 × 3 convolutional layer was applied at the end of the decoding. This refinement path can further improve the performance of output images and remove the artifacts at a high spatial resolution.^[^ ^34^ ^]^

### 2.3 Deep Learning Implementation

During training, the images were randomly split in a ratio of 8:2 for training and validation. The 3DFD U‐net network training was conducted on a workstation equipped with AMD Ryzen 9 3950X CPU, 128 GB RAM, and two NVIDIA GTX 3090 graphic cards. For the hyperparameters, we used the Adam optimizer to minimize the mean squared error loss with an initial learning rate of 1e‐4 and a batch size of eight for 300 epochs along with early stopping with a patience value of 10 which resulted in training ending at 213 epochs. The total trainable parameters are 6052025. For the batch normalization, the momentum parameter was set to 0.99 and the epsilon is 0.001. The loss function was the mean squared error of the intensity between the last layer output and ground truth per batch. To optimize training for speed and memory usage we utilized mixed precision training^[^ ^35^ ^]^ which harnesses the native ability of GPUs to perform calculations using float16 values by casting weights, activations, and gradients of the model to 16‐bit floats. This smaller representation allows for the model to be loaded and trained with effectively half the memory and bandwidth normally required, which in turn, lets us use larger batch sizes without sacrificing precision since a 32‐bit master copy of weights is maintained to recover gradients smaller than 2^−24^ (approximately 5%). Similar to,^[^ ^36^, ^37^ ^]^ we also implement a custom data loader using the *Keras.utils.Sequence* interface of Tensorflow 2 that yields batched volumes at training time via a thread‐safe generator instead of preallocating GPU memory of the size of the total dataset. This lets us train with a larger dataset than can be fit in GPU VRAM, in theory facilitating training with an infinite number of samples—realistically limited by the storage and model's representational capacity. The increase in batch size from four to eight that mixed precision affords speeds up training and lets the model learn from more data in the same amount of time. The change to data loading means we are no longer limited to 2400 samples that the 24 GB each GPU's VRAM can fit, and instead only load 8 samples per training step. While the overhead of reading from disk and copying input tensor batches to the GPUs for each step is not insignificant, the performance gain from using mixed precision offsets this with the effective time spent to process one volume going down from ≈450 to ≈250 ms owing to greater parallelization from the larger batch size. Furthermore, the value of exposing the model to 600 additional samples is reflected in the final validation mean‐squared error (MSE) loss of 2.56 (Figure S1, Supporting Information). All networks were implemented using Python 3.7 with a TensorFlow 2.6 backend. Total training took 35.5 hours.

### 2.4 Training Data Generation

We developed an effective simulation approach where the PA detection mechanism closely mimics the experimental transducer array, as shown in the data generation block of Figure 1. We used the MATLAB‐based acoustic simulation toolbox, K‐wave, to generate PA sinograms in 3D space.^[^ ^38^ ^]^ All 3D vasculature images were created using the Insight Segmentation and Registration Toolkit (ITK).^[^ ^39^ ^]^ The simulated vessels have diameters ranging from 0.1 to 4 mm,^[^ ^40^ ^]^ which correspond to primary features detectable by our linear array PAT system.^[^ ^41^ ^]^ Each 3D vasculature matrix has a size of 30 × 86 × 50 mm^3^ along axial, lateral, and elevation directions, respectively, with a pixel size of 0.1 mm. The vasculature matrix was placed 30 mm away from the transducer surface to mimic experimental scenarios. A linear transducer array with 128 curved elements was defined at the top of the simulation environment. To preserve imaging features, the transducer properties, including element height, receiving angle, focal zone, and frequency response, were defined based on the actual imaging system. To mimic scanning, the transducer array moved along the elevation direction (marked by the orange arrow in Figure 1) in the 3D environment at 0.2 mm step size. To improve the computing efficiency, for each scanning, the simulated region moved along with the array, as shown in the yellow region in Figure 1 data generation block. The size of the simulated volume for each scanning step is 30 × 86 × 15 mm^3^ along axial, lateral, and elevation directions, respectively. Each transducer element was composed of several voxels that received signals separately, and these signals were further combined into an A‐line signal belonging to the corresponding transducer element. Then, A‐line signals from all transducer elements were stacked in order along the lateral direction to form a 2D sinogram, which represents raw data acquired at one scanning location. We then used 2D‐stack reconstruction to generate input images for the network. In 2D stack reconstruction, we first used back projection to reconstruct a cross‐sectional image for each scanning position.^[^ ^42^ ^]^ The reconstructed 2D images were then stacked into a 3D matrix based on their spatial positions during scanning. EMI and system noise acquired from the experimental system was then applied to the simulated 3D matrix. After that, the data was divided into several cubes with 128 pixels along each dimension as the network input (shown in Figure 1 validation block). The position of each cube in the 3D matrix was recorded so that they could be combined into the 3D matrix again after processing.

### 2.5 Image Fusion for In Vivo Data Enhancement

As each 3D cube was processed separately in the learned model, there could be slight misalignments at the cube boundaries when they were re‐combined (Figure S2a, Supporting Information). To improve the continuity, we developed an image fusion method for the output data. In this method, a normalized nonlinear weighting map (M) was applied to the boundary region of adjacent matrices.^[^ ^43^ ^]^ As shown in Eq. 1, the map contains an exponential function that defines the weighting based on the position. Here, *x* represents the index of the matrix M along lateral and elevation, respectively. A is the width of the overlaid regions, which is 32 in most of our data (the cube size is 128^3^).

(1) $$M\left(x\right)=\frac{e^{x/A}-1}{e-1}1\leq x\leq A$$

The maps were applied during image fusion to eliminate sudden changes at the edge of the cubes. Therefore, the misalignment among matrices was eliminated and the image continuity was improved. The images before and after fusion are shown in depth‐encoded MAP (maximum amplitude projection) images in Figure S2a,b, respectively.

## 3 Results

### 3.1 Numerical Validation

**Figure 2:**
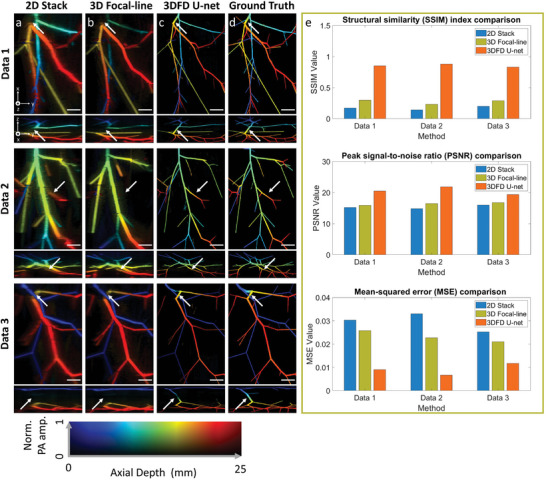
Network performance evaluation using numerical data. a) (top to bottom) Three sets of input data generated by the 2D stack reconstruction. For each set, the top view is shown at the top and the cross‐sectional view is shown at the bottom. b) The same set of data generated by 3D focal‐line reconstruction. c) Output images from 3D fully‐dense (3DFD) U‐net. d) Ground truth of the three datasets. e) Bar charts of structural similarity index measure (SSIM), peak signal‐to‐noise ratio (PSNR), and mean‐squared error (MSE) quantification among three approaches (top to bottom). Scale bar: 10 mm. *x*, *y*, and *z* denote the lateral, elevation, and axial directions of the transducer array, respectively.

We first validated the performance of our algorithm in three sets of numerical data. None of these data were used in training. For easy comparison, all images were normalized and shown in depth‐encoded MAP images along either lateral and elevation directions (top view) or axial and elevation directions (cross‐sectional view). The colors from blue to red represent locations from shallow to deep, starting from the reconstruction depth (30 mm from the transducer array surface). The other axis in the color map represents the normalized PA intensity. This representation allows us to reproduce both PA intensity and signal depth in a 2D image. All depth‐encoded images in the following sections are presented in the same manner. **Figure** **2**a–c shows images reconstructed by the 2D stack, 3D focal‐line,^[^ ^44^ ^]^ and 3DFD U‐net algorithms, respectively. Figure 2d displays the ground truth image for comparison. As 2D stack reconstruction does not explore the connection among frames, the reconstructed image looks blurry along the elevation direction, and the background noise is also obvious. Due to the poor elevation resolution, it can be seen that the vessels extended along the lateral direction exhibited a thicker size compared to their elevational counterparts. The 3D focal‐line algorithm slightly improves the elevation resolution based on the virtual detector concept. However, it still cannot recover the true size of the vasculature. Moreover, both methods are affected by the limited view problem. For instance, vessels indicated by white arrows in Figure 2 extend along the axial direction and is not fully visible in Figure 2a,b. In comparison, outputs from 3DFD U‐net provide significant improvements in terms of improved image resolution and reduced noise level. Vessels in 3DFD U‐net output are also displayed more continuously. In addition, features distorted by the limited view problem can be partially recovered, addressing a major issue in linear array‐based PAT. It can be observed that vessels indicated by white arrows extend to the deep region rapidly and therefore, cannot be fully detected by the linear transducer array (Figure 2a). In comparison, 3DFD successfully recognized these vessels from the input and restored them in the processed image (Figure 2c). Similar improvements can also be observed from the in vivo validations presented in the next section.

We also quantitatively evaluated the quality of outputs from two algorithms by calculating the structural similarity index measure (SSIM), the peak signal‐to‐noise ratio (PSNR), and MSE of each image. The ground truths in Figure 2d denote the references used for calculation. The bar chart featuring the quantified values can be found in Figure 2e. We can see that the output image of 3DFD U‐net provides higher SSIM, PSNR, and MSE. These indicated that our deep learning algorithm could reconstruct high‐quality images with higher resolution than the conventional 2D stack image.

### 3.2 Phantom Validation

**Figure 3:**
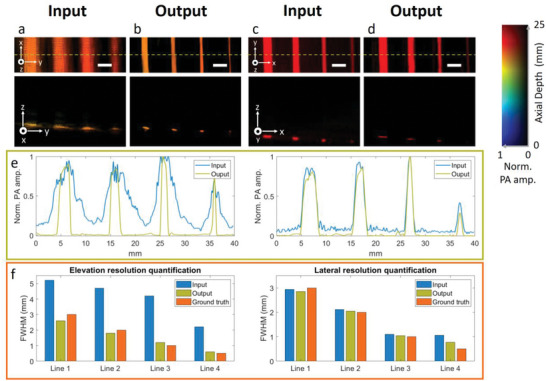
True object size recovery validation using printed phantom placed along lateral and elevation directions. a) Input images of lines extended along the lateral direction in the top view and cross‐sectional view (along the yellow dashed line in top row). b) Output images of corresponding sections of (a). c) Input image of lines extended along elevation direction in the top view and cross‐sectional view (along the yellow dashed line in top row). d) Output image of corresponding sections of (c). e) Left panel: Intensity profiles acquired from cross‐sectional images in (a) and (b); Right panel: Intensity profiles acquired from cross‐sectional images in (c) and (d). f) Left panel: Elevation resolution quantified from input, output, and ground truth. Right panel: Lateral resolution quantified from input, output, and ground truth. Scale bar: 5 mm. *x*, *y*, and *z* denote the lateral, elevation, and axial directions of the transducer array, respectively.

We then tested the performance of the trained network in phantom. In this test, we verified whether the algorithm could restore the true size of the object instead of simply shrinking it. The phantom was made with a transparent sheet printed with four black lines with diameters of 0.5, 1.0, 2.0, and 3.0 mm, respectively (Figure S3, Supporting Information). The phantom was placed 50 mm away from the transducer array surface, which is out of the transducer's focal zone. We first placed the phantom parallel to the transducer and then rotated it 90 degrees in the second scanning. The two experiments allow us to evaluate the performance along the elevation and lateral directions of the transducer, respectively. The top row of **Figure** **3**a,c shows the depth‐encoded 2D‐stack images for the phantom placed in two orientations. The bottom row of Figure 3a,c provides the cross‐sectional images along the yellow dash‐line in the top row. Due to the poor elevation resolution, the four lines look much wider in Figure 3a. In comparison, the lines in Figure 3c look sharper as the system's lateral resolution is better than that of elevation. However, the 0.5‐mm line still looks slightly wider than its true size as it is smaller than the lateral resolution. Figure 3b,d showcases the output images from the network and their corresponding cross‐sectional images along the yellow dash lines. It can be seen that the line widths in Figure 3b are significantly reduced due to the improved elevation resolution, while those in Figure 3d remain unchanged. The left and right panels of Figure 3e illustrate the PA intensity profile along the dashed lines in Figure 3a–d, respectively. Blue and green curves represent the input and output intensity profiles, respectively. We further quantified the object size along lateral and elevation directions based on the full width at half maximum (FWHM). The results are summarized in Figure 3f. It can be seen that the output diameters were similar to that of the ground truth along both elevation and lateral directions.

### 3.3 In Vivo Validation

Following promising results from numerical and phantom validation, we deployed the 3DFD‐Unet to the in vivo data to further validate its performance.

**Figure 4:**
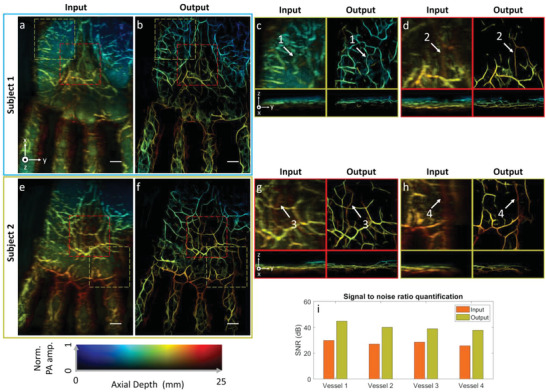
Validation in human palm data. a,e) Depth‐encoded input image reconstructed by 2D stack reconstruction. b,f) Output image of (a) and (e), respectively. c) Top and cross‐sectional views of regions marked by a yellow dashed box from input and output images of subject 1. d) Top and cross‐sectional views of regions marked by a red dashed box from input and output images of subject 1. g) Top and cross‐sectional views of regions marked by red dashed box from input and output images of subject 2. h) Top and cross‐sectional views of regions marked by yellow dashed box from input and output images of subject 2. i) signal‐to‐noise ratio (SNR) quantification of four vessels from input and output images. Scale bar: 10 mm. *x*, *y*, and *z* denote the lateral, elevation, and axial directions of the transducer array, respectively.

**Figure** **4**a,e demonstrates MAP images of human palms of two subjects reconstructed by the 2D‐stack algorithm. Due to strong skin surface signals and reconstruction artifacts, vasculature underneath the skin layer cannot be visualized. Also, the vascular structures were exhibited in a discontinuous manner, which is anatomically inaccurate. In addition, the poor elevation resolution makes it hard to reveal vessels extended along the lateral direction. Figure 4b,f denotes enhanced output images from our algorithm of two subjects, respectively. Due to improved resolution, finer vasculatures can be observed in all directions. It can also be seen that most skin surface signals were removed by the algorithm, allowing for the visualization of deep vessels. While the skin surface signals were not introduced in the training data, they were still removed because the skin features look different than vasculature and thus were treated as noise by the algorithm. For better illustration, we selected two regions (marked in yellow and red dashed frames) from each data and plotted the enlarged and cross‐sectional views in Figure 4c,d,g,h. It can be seen from the input image that vasculature was not exhibited clearly due to the low spatial resolution and skin artifacts images. In comparison, finer vascular structures can be visualized from output images. The bottom row of Figure 4c,d,g,h demonstrates the cross‐sectional images. It can be seen that deep vessels are more visible in the output images and the vessel discontinuity caused by the limited view problem is reduced. A detailed comparison of skin removal using different approaches can be found in Figure S4 (Supporting Information). To further verify our algorithm's performance, we chose various vessels and quantified the signal‐to‐noise ratios (SNR). Figure 4i demonstrates the SNR values of input and output images from each vessel. As shown in the bar chart, vessels from output images exhibit higher SNR compared to the input. In general, output images achieved remarkable improvement in different aspects, exhibiting clear and continuous vasculature. We also conducted an imaging study on the human forearm, where similar improvements can be observed. Details can be found in Figure S5 (Supporting Information).

### 3.4 Application Case Studies

#### 3.4.1 3D Finger Vein Biometrics Resilience Analysis

**Figure 5:**
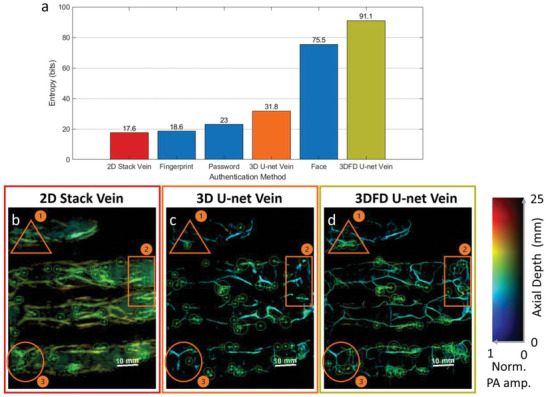
a) Entropy quantification of different biometrics authentication approaches. b–d) Photoacoustic (PA) images of the same palm and the extracted vein biometric feature points. b) 2D stack; c) 3D U‐net; d) 3D fully‐dense (3DFD) U‐net.

First, we validate the 3DFD U‐net potential for biometrics authentication. Biometrics entropy (BE) has been widely adopted in evaluating the security performance (capacity) of authentication systems (e.g., password and face authentication).^[^ ^45^ ^]^ It measures the extra certainty that the authentication system provides to identify a specific person from all the people in the world. The more certainty a system can provide, the higher security this system maintains. Recent studies explored utilizing the unique palm vein structure captured by a photoacoustic sensor for biometric authentication.^[^ ^46^, ^47^ ^]^ Given the better performance of 3DFD U‐net compared to 2D stack and 3D U‐net, we anticipate that the extra details of vein structure provided by 3DFD U‐net can provide more security for this application, and BE can quantitatively measure this improvement. To validate our hypothesis, we use the same method stated in^[^ ^48^ ^]^ to extract the vein structure features and examine the entropy of the vein structure features captured by the three mentioned PA Vein methods. The results are shown in the bar chart (**Figure** **5**a) below. We observe 3DFD U‐net approach carries much more identity information compared with the 2D stack and 3D U‐net methods. The increase of biometrics entropy is around 73.5 and 60 bits, respectively. This indicates that the vein structure details revealed by the proposed novel 3DFD U‐net approach are highly related to the critical identity information and can bring extra security to the system based on the proposed system. With the help of significant entropy improvement, the security level of palm vein biometrics surpasses the level of face biometrics, which is a widely used biometrics in consumer electronics. As shown in Figure 5b–d, we observe three representative areas in the vein feature visualization. In areas 1 and 2, we can see the 3DFD U‐net dramatically improves the clarity of vein imaging, thereby providing more identity information that can be captured by the feature extraction algorithm, i.e., more feature points. In area 3, by comparing the features of 3D U‐net and 3DFD U‐net, we can see 3DFD U‐net reveals veins in deeper tissues, which brings extra identity information as captured.

#### 3.4.2 Imaging of Foot Ulcer

**Figure 6:**
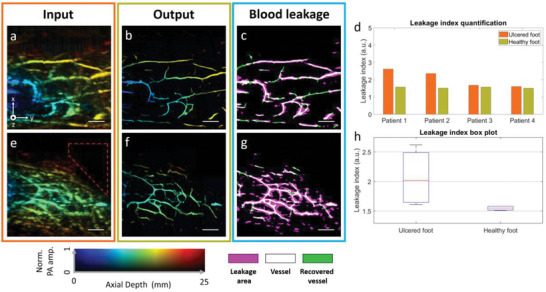
Photoacoustic imaging of foot ulcer. a,e) Input images of the healthy and ulcered foot, respectively. b,f) Output images of (a) and (e), respectively. c,g) Blood leakage quantification from healthy and ulcered foot, respectively. d) Leakage index quantification on four subjects. h) box plot of quantified leakage index from ulcered and healthy foot. Scale bar: 10 mm. *x*, *y*, and *z* denote the lateral, elevation, and axial directions of the transducer array, respectively.

In this study, we imaged the foot vasculature of patients with peripheral artery diseases (PAD) and chronic foot ulcers. The goal is to identify whether the enhanced vascular images provided a better clinical assessment of tissue perfusion and identification of leaky vessels (increased endothelial permeability).^[^ ^49^, ^50^ ^]^ Images shown in **Figure** **6** came from a patient with ulcers on the right foot and amputation of the big toe (marked with the red dashed zone), while the left foot looked normal. Figure 6a,b displays the original and processed PA depth‐encoded MAP images of the healthy foot, while Figure 6e,f shows the images of the ulcered foot. The proposed neural network significantly improves image quality by enhancing vessel continuity and accurately recovering the vessel size.

Leakage quantification is an important aspect in evaluating the perfusion condition of subjects with chronic wounds, as poor blood circulation often leads to leaking vessels in these areas.^[^ ^51^ ^]^ However, automatically quantifying leakage based on PA images is challenging, especially in the absence of ground truth vessel information for in vivo images. Here, we capitalized the enhanced vascular image provided by 3D FD UNet to quantify the leakage. The algorithm involves binarizing the input (Figure 6a) and output (Figure 6b) PA images and counting the total number of non‐zero pixels in each binarized image. The leakage index is then computed by the ratio of pixel numbers in the input and 3D FD UNet enhanced images. A higher index thereby indicates a blurrier vessel due to leakage. Figure 6c,g presents a comparison of the binarized images of the two feet. The ulcered foot exhibits a larger leakage area (indicated in pink). Additionally, the neural network successfully recovered the vessels affected by the limited view problem (marked in green).

The model and proposed algorithm were tested on four subjects (two with ulcers on the left foot and two with ulcers on the right foot), and the quantification results are summarized in Figure 6d,h. All subjects included in our study had peripheral artery disease (PAD) and toe amputation (Table S1, Supporting Information). The leakage indices of the ulcered feet were consistently higher than those of the healthy feet, and the box plot analysis revealed a larger variance in the leakage index for the ulcered feet compared to the healthy feet. This variability is expected due to individual differences in condition. These results demonstrate that the proposed model effectively recovers vessels, enabling quantitative estimation of vessel leakage.

#### 3.4.3 Imaging of Breast Cancer

**Figure 7:**
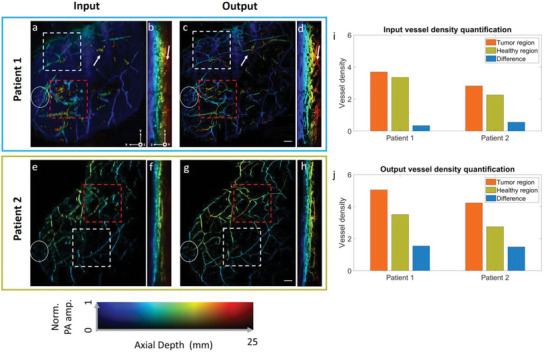
In vivo validation on malignant human breast data. a) Patient 1 input image of the breast presented in frontal view. The red dashed box indicates the tumor region, while the white dashed box indicates a nearby healthy region. The white circle marks the nipple. b) Patient 1 input image of the breast presented in cross‐sectional view. c,d) Corresponding 3D FDUNet output images of (a) and (b), respectively. e) Patient 2 input image of the breast presented in frontal view. f) Patient 2 input image of the breast presented in cross‐sectional view. g,h) Corresponding 3D FDUNet output images of (e) and (f), respectively. i) Input vessel density quantification results from two patients. j) Output vessel density quantification results from two patients. Scale bar: 10 mm. *x*, *y*, and *z* denote the lateral, elevation, and axial directions of the transducer array, respectively.

The last case study is the imaging of breast cancer. As the progression of breast cancer is often associated with angiogenesis,^[^ ^52^ ^]^ the vascular features captured by PA imaging have good potential for tumor screening and diagnosis.^[^ ^8^, ^53^ ^]^ In **Figure** **7**a,b, we presented breast image from a patient volunteer with dark skin color and a breast cup size of D. Based on the clinical report, her left breast has two tumors. One is 3 × 3 × 4 mm^3^ in size and is located at 3 o′clock and 2 cm from the nipple. The other is 6 × 4 × 3 mm^3^ in size, located at 4 o′clock and 2 cm from the nipple. The tumor subtype is luminal A, which is associated with prominent external vessels in PA images and hence high vessel density.^[^ ^54^ ^]^ The nipple region was marked by a white circle in Figure 7a,c. As the images were presented in the frontal view, we were able to locate the suspicious regions based on the clinical report and mark the area with a red dashed box. As the patient has a dark skin color, the breast vessels were shadowed by the skin contrast, as indicated by the white arrow in Figure 7a,b. These factors made it challenging to clearly visualize the breast's vascular structure. Figure 7c,d denotes the output of the 3DFD U‐net. The skin absorption signals were effectively removed, revealing the vessels in the deep tissue. We observed that the system noise and skin absorption contaminated the vascular structures in the red‐dashed box in Figure 7a, making it difficult to visualize the tumor features. In contrast, the region framed by the red‐dashed box in Figure 7c indicated that our algorithm clearly revealed vessels from deep regions. Hypervascularity can now be clearly observed in the tumor regions. We then quantified the vessel density of the malignant region (red‐dashed box) and a normal breast region (white‐dashed box) as the vessel density has been reported to correlate with breast malignancy.^[^ ^55^ ^]^ Similarly, we employed the same methodology to assess the vessel density in another patient with the same tumor subtype (Luminal A). According to the clinical report, the cancer lesion is located at 2 o′clock and 12 cm from the nipple. The input and output images can be seen in Figure 7e–h, respectively. Enhancement in vessel contrast can also be clearly observed in this case. The vessel density quantification results from input and output images are summarized in Figure 7i,j, respectively. The healthy region (white box) was selected close to the tumor region. The resulting images exhibit significantly enhanced contrasts in vessel density between the tumor region and the healthy region.

To verify whether the algorithm assists in quantifying the whole‐breast vessel density, we selected four patients with different breast cup sizes and tumor subtypes. Detailed information of these patients can be found in Table S2 (Supporting Information). Although the local vascular features may differ among different tumor subtypes, it is anticipated that the breast affected by the tumor will exhibit a higher vessel density compared to the contralateral healthy breast, owing to tumor‐associated angiogenesis.^[^ ^56^ ^]^ The vessel density quantification results can be found in Figure S6 (Supporting Information). The quantified vessel density is the global vessel density from the whole breast. Upon analyzing the output images, we observed a notable contrast in vessel density between the tumor‐bearing breast and the contralateral healthy breast. This result indicates that the 3D FD Unet can be used to improve photoacoustic breast cancer screening and diagnosis.

## 4 Discussion

In this study, we designed a 3D simulation method to generate the numerical photoacoustic raw data and applied a 3D neural network to enhance the volumetric image quality on linear array based PAT. The training network was developed based on the fully dense U‐net,^[^ ^30^ ^]^ which has been widely used in biomedical imaging applications with great performance.^[^ ^27^, ^29^, ^57^ ^]^ Compared to traditional 2D neural network approaches, 3D‐trained networks can better reveal the vascular structure in all 3D planes by exploring the data's volumetric information rather than cross‐sectional images. Compared with the conventional 3D U‐net network, the 3D FD U‐net leveraged the benefit of dense connectivity in each layer, allowing it to learn additional features and improve the model performance. A detailed comparison of different reconstructed approaches (2D stack, Deep‐E, 3D U‐net, and 3DFD U‐net) can be found in Figures S7 and S8 (Supporting Information). Moreover, we overcame the memory allocation and efficiency problems in 3D network training by utilizing mixed precision training, which allowed for a larger batch size of eight (data matrix size is 128 × 128 × 128) and avoided training crashes. The addition of dense connectivity aids with better propagation of information through the network. It makes the architecture more robust to vanishing gradients,^[^ ^29^ ^]^ allowing us to train a single, compact, and end‐to‐end model reliably converging even at full resolution. Therefore, we did not need to utilize other complex 3D training techniques, such as progressive growing, where the model must be trained iteratively from lower to higher resolutions with multiple subnetworks for each resolution, and each subnetwork needs to have its own hyperparameters like batch size, as implemented in.^[^ ^18^ ^]^

To validate the performance of the 3DFD U‐net network, we tested the trained model on the simulated volumetric vascular matrix, printed line phantoms, and in vivo hand, palm, foot, and breast data. The numerical data demonstrates that our technique can significantly improve image quality with an average SSIM of 0.85 (versus 0.17 in input) and an average PSNR of 20.48 (versus 15.18 in input). We also used printed line phantoms to verify the recovery object size along elevational and lateral directions. The FWHM quantification proved that the trained 3DFD U‐net model improved the resolution of the image, up to the best native resolution of the transducer (0.5 mm for lateral and 0.6 mm for the elevational). In addition to resolution improvement, the background noise was eliminated. After phantom validation, we could finally apply the trained 3DFD U‐net to human clinical data, where we were able to generate clear 3D vascular images of the palm, breasts, and feet of humans. The in vivo data indicates that the 3DFD U‐net improves vessel contrast and continuity. More importantly, the limited‐view problem of the linear array was resolved, allowing vessels perpendicular to the transducer to be visible. This is because our technique learned the vascular structure in the 3D domain and explored the connection among different imaging planes to restore the missing features. These enhanced vascular images allow us to demonstrate the versatility of 3DFD U‐net in a range of human imaging applications. These include achieving greater entropy for biometric identification through finger vein patterns, ensuring more reliable quantification of leaky vessels in the context of foot ulcers, and simplifying the detection of hypervasculature in breast cancer cases. Such compelling outcomes suggest that our algorithm is well‐suited for clinical photoacoustic imaging. It should be noted that while the current study utilized a 1064 nm wavelength, the technique can be applied to PA systems with other wavelengths, as our K‐wave simulation algorithm utilized general light absorption by hemoglobin without relying on absorption coefficients specific to a particular wavelength. The prospect of multiwavelength imaging might offer new possibilities in multicontrast enhancement for better tissue assessment and disease diagnosis.

Compared to existing 3D neural network studies in photoacoustic imaging, our work utilized fully sampled linear array data, while others utilized sparsely sampled data from cylindrical^[^ ^23^ ^]^ or hemispherical arrays,^[^ ^18^ ^]^ which have a smaller data size. In addition, due to the limited‐view problem, it is more challenging to acquire in vivo ground truth images in linear arrays. We addressed these issues through a precise simulation algorithm that mimics the experimental situation. Combined with the more efficient training approach, we were able to utilize the largest 3D dataset (3000 matrices with dimensions of 128 × 128 × 128) in photoacoustic imaging neural network training. We envision that our proposed algorithm will be a valuable tool for photoacoustic research and clinical imaging.

## 5 Conclusion

In this study, a 3DFD U‐net was introduced to enhance volumetric vascular imaging performance in linear array based photoacoustic tomography. To train this network, we developed an accurate simulation model with real experimental noise to mimic volumetric vascular images acquired by linear transducer arrays. The optimized data loading/streaming method and mixed precision training enhanced the performance and efficiency of the neural network. The trained model was successfully validated through phantoms and various in vivo images. The results demonstrated that our algorithm improves spatial resolution, reduces image noise, refines vessel continuity, and reveals deeper and limited‐viewed vessels. Overall, we proposed a novel deep learning approach to enhance photoacoustic images beyond the capabilities of traditional 2D methods, and we have successfully applied this method to a range of human imaging applications. As PAT‐based vascular imaging is a relatively new field undergoing active exploration in clinical applications,^[^ ^58^, ^59^ ^]^ we envision that our proposed algorithm would further advance the field and improve disease screening, diagnosis and treatment monitoring.

## Conflict of Interest

J.X. is the founder of Sonioptix, LLC, which, however, did not support this work. All other authors declare no conflict of interest.

## Supporting information

Supporting Information

## Data Availability

The data that support the findings of this study are available from the corresponding author upon reasonable request.
