# Supplementary material for: Deep Learning Enhanced Volumetric Photoacoustic Imaging of Vasculature in Human
Source: Adv Sci (Weinh). 2023 Aug 2;10(29):2301277. doi: 10.1002/advs.202301277 (PMC10582405; doi:10.1002/advs.202301277)
Supplement: Supplementary file 1 — Supporting Information [file ADVS-10-2301277-s001.pdf]

ADVANCED SCIENCE
Open Access

Supporting Information

for *Adv. Sci.*, DOI 10.1002/advs.202301277

Deep Learning Enhanced Volumetric Photoacoustic Imaging of Vasculature in Human

*Wenhan Zheng, Huijuan Zhang, Chuqin Huang, Varun Shijo, Chenhan Xu, Wenyao Xu and Jun Xia**

Deep Learning Enhanced Volumetric Photoacoustic Imaging of Vasculature in Human

Wenhan Zheng [1,γ], Huijuan Zhang [1,γ], Chuqin Huang [1], Varun Shijo[1,2], Chenhan Xu[2], Wenyao Xu[2], Jun Xia [1,2]*

1 Department of Biomedical Engineering, University at Buffalo, The State University of New York, Buffalo, New York, NY 14260, USA
2 Department of Computer Science and Engineering, University at Buffalo, The State University of New York, Buffalo, New York, NY 14260, USA
* Correspondence: junxia@buffalo.edu
γ Equal contribution

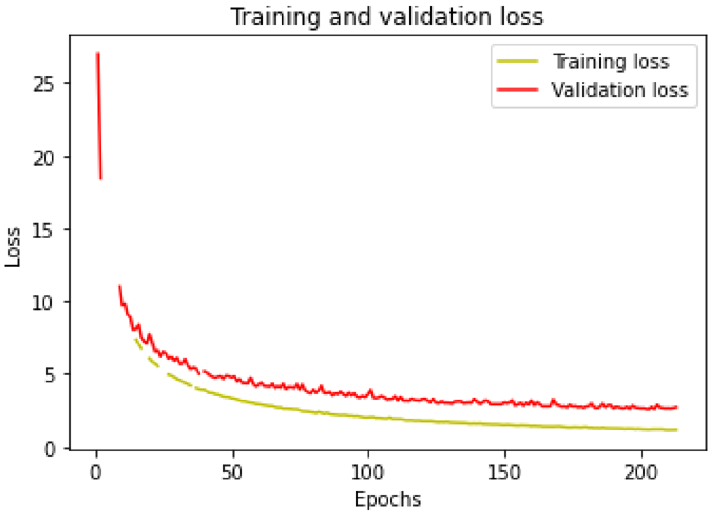

Supplementary Figure 1. Training and Validation loss plots vs. epochs. Discontinuities in the graphs can be attributed to exploding gradients resulting in loss being reported as NaN values.

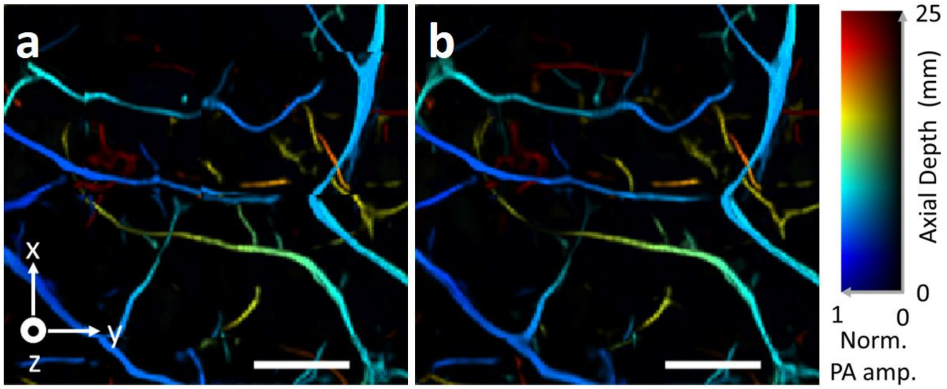

Supplementary Figure 2. Comparison of images before (a) and after (b) image fusion. Scale bar: 10 mm. x, y, and z denote the lateral, elevation, and axial directions of the transducer array, respectively.

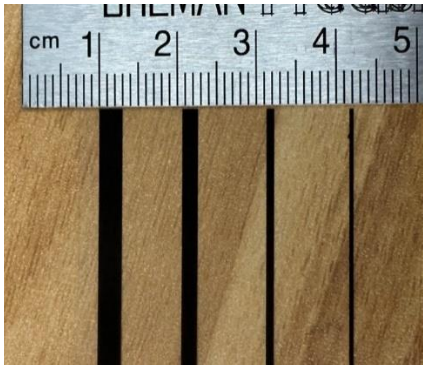

Supplementary Figure 3. Photo of printed phantom

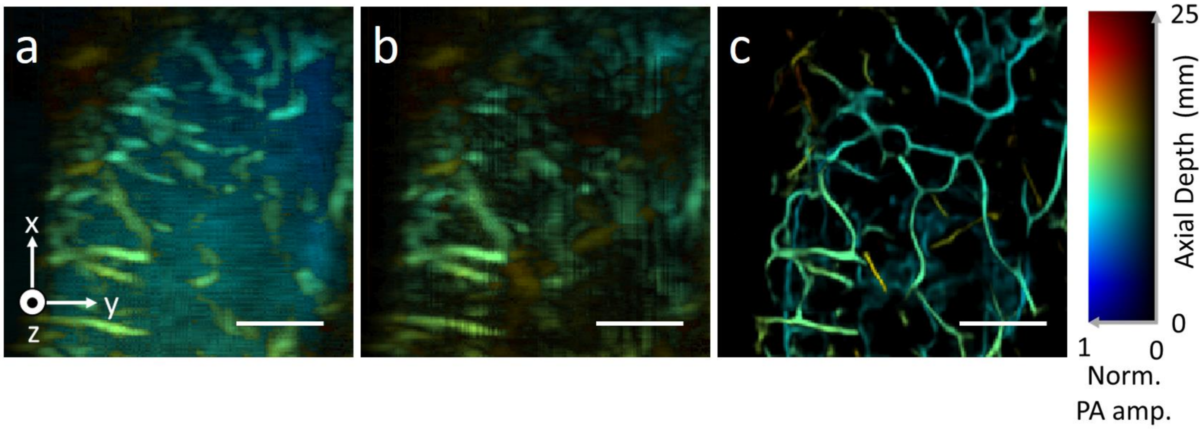

Supplementary Figure 4. Skin removal comparison using human palm data. (a) Original image. (b) Image after skin removal using Matlab-based algorithm. (c) Output image from 3DFD U-net. Scale bar: 10 mm. x, y, and z denote the lateral, elevation, and axial directions of the transducer.

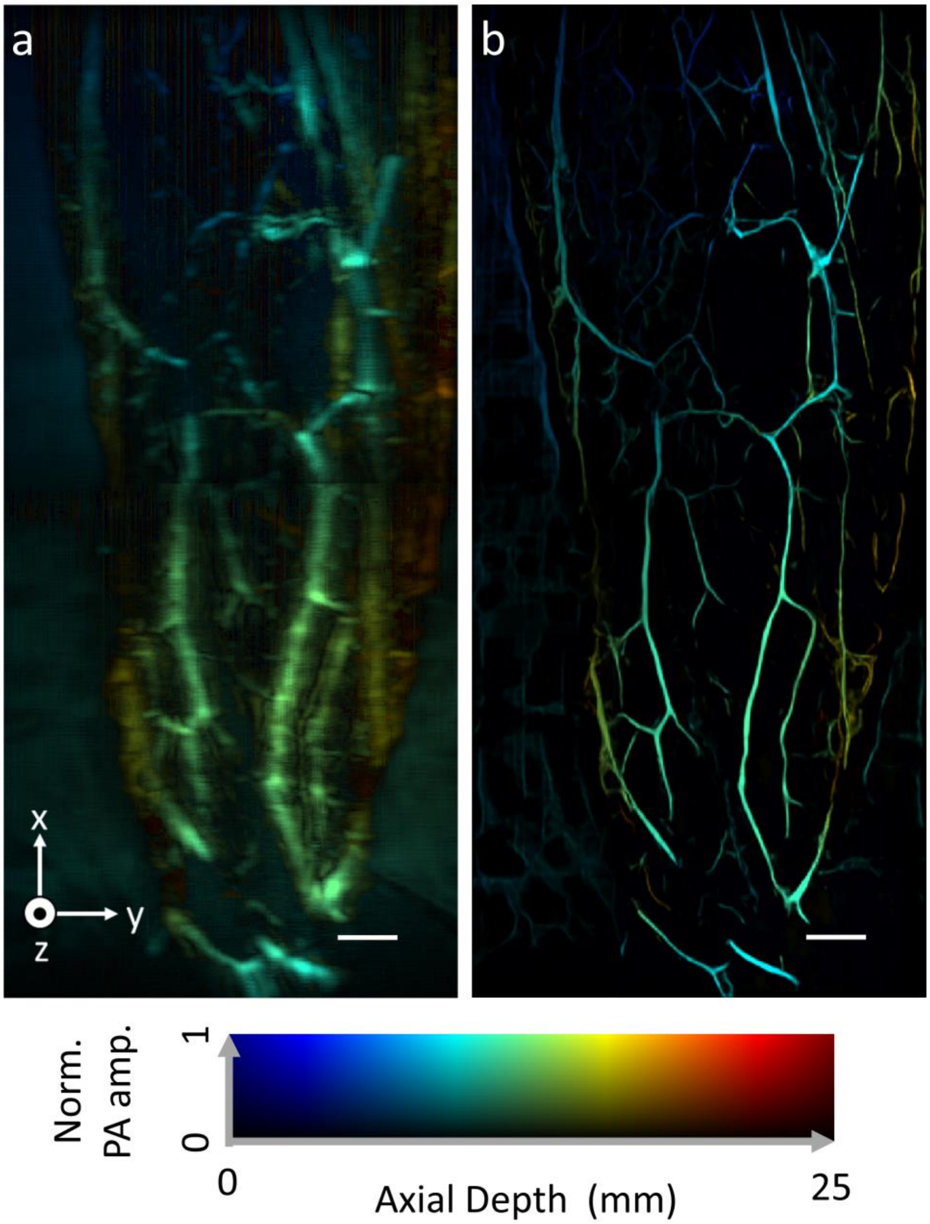

Supplementary Figure 5. In vivo validation using human forearm data. (a) Input image reconstructed by 2D stack reconstruction. (b) Output image of (a). Scale bar: 10 mm. x, y, and z denote the lateral, elevation, and axial directions of the transducer.

	Gender	Age	Race	Medical condition
Patient 1	Male	N/A	Indian	Amputation, big toe lost
Patient 2	Male	58	Spanish	Amputation, big toe lost
Patient 3	Male	52	White	Amputation, big toe lost, second toe abnormal
Patient 4	Female	N/A	White	Amputation, third to fifth toes lost

Supplementary Table 1. Information of patients with foot ulcer.

	Cup size	Skin color	Breast density	Tumor subtype
Patient 1*	D	Black	Heterogeneously dense	LUMA
Patient 2*	DDD	Black	Scattered fibroglandular dense	LUMA
Patient 3	B	White	Heterogeneously dense	LUMB
Patient 4	DDD	Medium	Heterogeneously dense	LUMB
Patient 5	A	Medium	Heterogeneously dense	/
Patient 6	D	White	Scattered fibroglandular dense	LUMA

Supplementary Table 2. Information of patients with breast cancer. *Patients in Fig. 7 of main text.

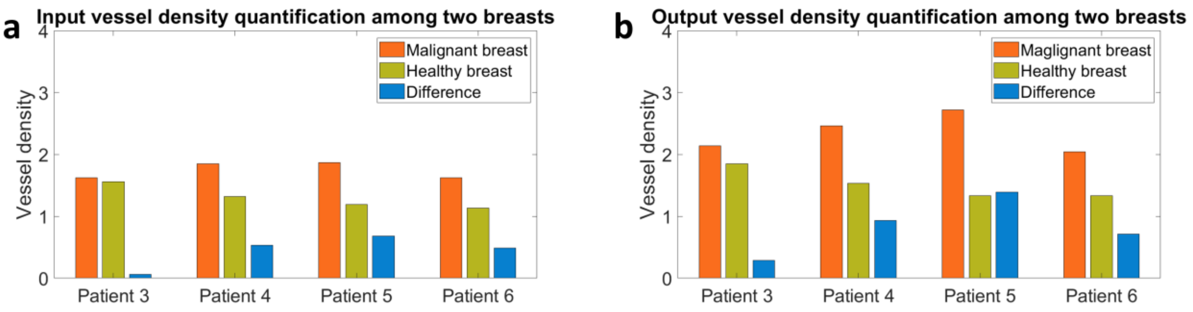

Supplementary Figure 6. Vessel density quantification from four patients. (a) Input vessel density quantification among malignant and healthy breasts. (b) Output vessel density quantification among malignant and healthy breasts.

Comparison of different reconstruction approaches

To further demonstrate the advantages of our technique, we also compare 3DFD U-net with the 2D stack reconstruction, Deep-E, and 3D U-net on both numerical and experimental data. Supplementary Figure 6 shows the MAP images of the numerical vessels reconstructed by four methods. SSIM and PSNR were quantified and the results proved that the 3DFD U-net gave the best performance. The ground truth image is shown in Fig 6a, while Fig 6b corresponds to the reconstruction obtained using the 2D stack approach. In contrast, Fig 6e shows the result obtained using our proposed 3DFD U-net method. Our results demonstrate that the 3DFD U-net can reveal more vessels than other methods. With the help of the ground truth image, we can confidently state that the new vessels are not fake features generated from the 3DFD U-net. Instead, they are signals that cannot be reconstructed in the 2D method due to the limited view problems. This indicates the reliability of our proposed network. Moreover, 3DFD U-net also outperformed the purely 3D U-net method. We then extend this comparison to experimental data and present the results below.

Supplementary Figure 7 demonstrates that the vessel contrast and sharpness are all enhanced by different neural networks. However, as Deep-E (Fig 7b) processed each cross-section independently, discontinuity can be observed in the output image. The 3D U-net (Fig 7c) processed the data in 3D and thereby revealed better vessel continuity. However, it cannot fully extract vessel information from 3D dimensions, as evidenced by the missing vessels (marked in the red dash box). In comparison, the 3DFD U-net (Fig 7d) revealed more vessels with a cleaner background and improved spatial resolutions. This comparison further validated the superiority of the 3DFD U-net network over other deep learning approaches.

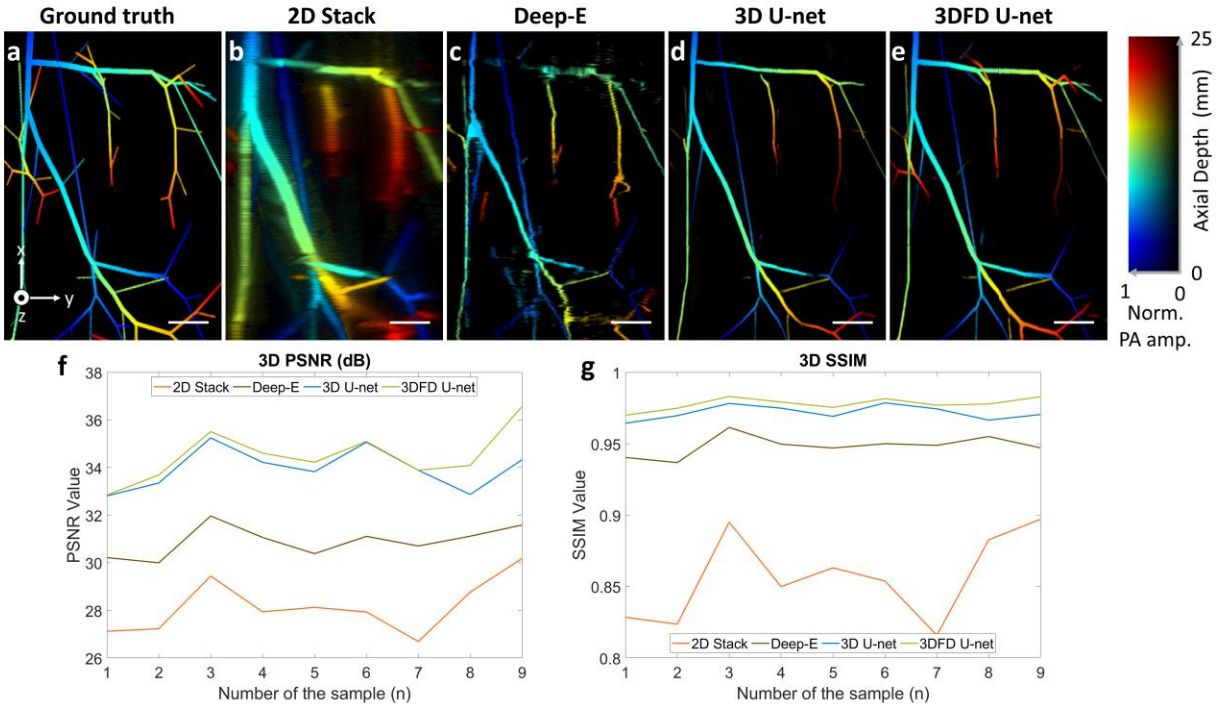

Supplementary Figure 7. Comparison of different reconstruction methods using a numerical vascular phantom. (a) Ground truth image. (b) Input image from 2D Stack reconstruction. (c) Output image from the Deep-E model. (d) Output image from the 3D U-net model. (e) Output image from the 3DFD U-net model. (f) Quantified 3D PSNR of different methods in nine test samples. (g) Quantified 3D SSIM of different methods in nine tested samples. Scale bar: 10 mm. x, y, and z denote the lateral, elevation, and axial directions of the transducer array, respectively.

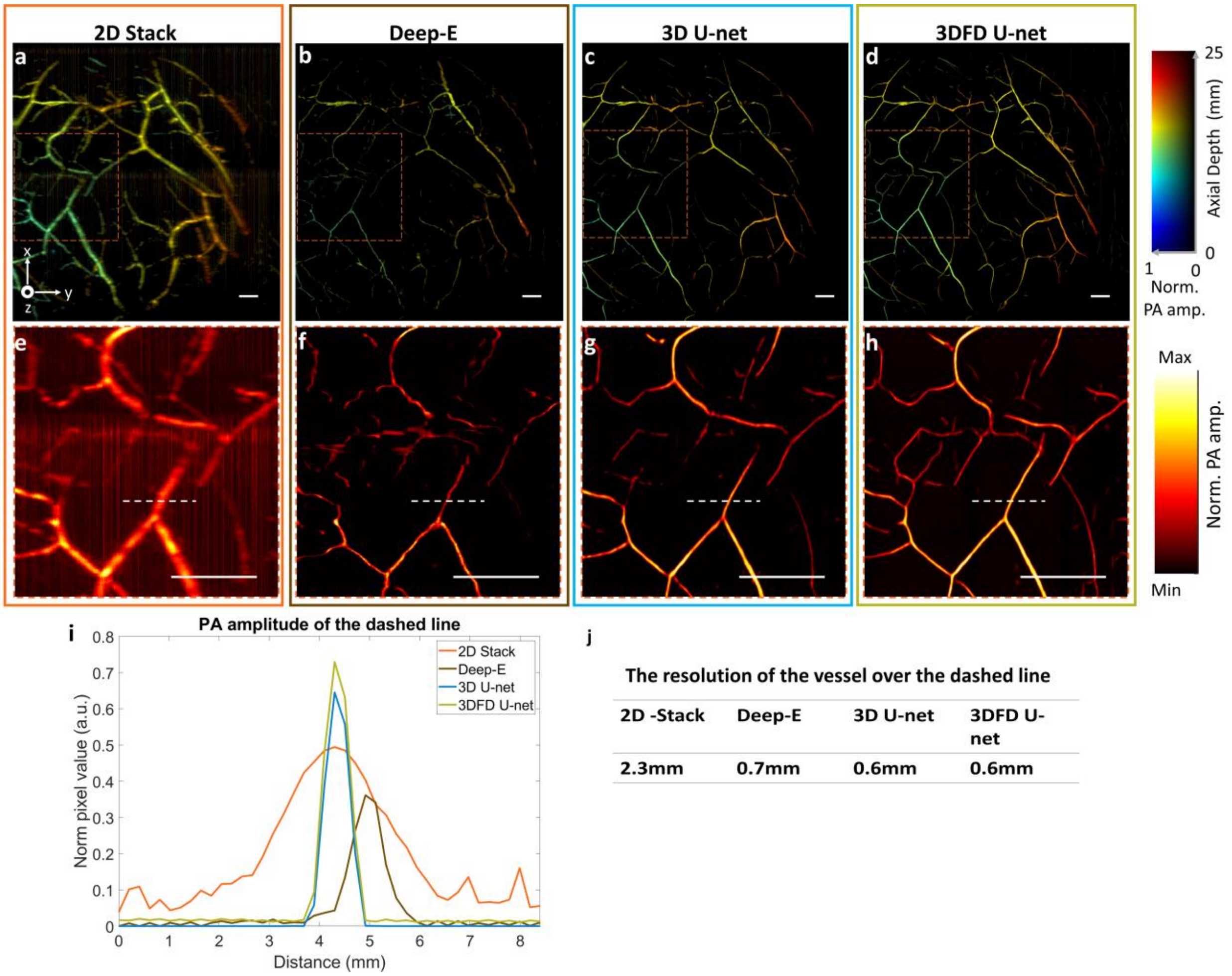

2D -Stack	Deep-E	3D U-net	3DFD U-net
2.3mm	0.7mm	0.6mm	0.6mm

Supplementary Figure 8. Performance of reconstruction approaches quantified based on human breast data. (a) Input image by 2D stack reconstruction. (b) Output image from Deep-E. (c) Output image from 3D U-net. (d) Output image from 3DFD U-net. (e), (f), (g), (h): Close-up view of regions outlined by the red dashed box in the corresponding upper row. (i) Profiles of the PA amplitude along the white dashed line in e-h. (j) FWHM quantification of the breast vessel marked by the white dashed line in e-h. Scale bar: 10 mm. x, y, and z denote the lateral, elevation, and axial directions of the transducer array, respectively.
